# A case of kerion celsi caused by *Trichophyton tonsurans* with dermatophytid reaction mimicking a drug eruption and endothrix infection confirmed in pathological tissue

**DOI:** 10.1016/j.mmcr.2024.100691

**Published:** 2024-12-25

**Authors:** Makoto Ishiai, Hiroshi Tanabe

**Affiliations:** Department of Dermatology, Tenri Hospital, 200 Mishima, Tenri, Nara, 632-8552, Japan

**Keywords:** Kerion celsi, *Trichophyton tonsurans*, Dermatophytid reaction, Endothrix infection

## Abstract

We report a case of kerion celsi caused by *Trichophyton tonsurans* in a teenage male judo athlete, presenting with a lesion in the occipital region. Following the initiation of systemic antifungal therapy, the patient developed a dermatophytid reaction, necessitating differentiation from a drug eruption. Direct microscopy of the affected area confirmed the presence of fungal elements, and histopathological examination revealed endothrix invasion, supporting the continuation of treatment. A drug-induced lymphocyte stimulation test for terbinafine, conducted post-treatment, was negative. This case highlights the importance of distinguishing dermatophytid reactions from drug eruptions to ensure uninterrupted antifungal therapy.

## 1 Introduction

Since the early 2000s, *Trichophyton tonsurans* has emerged as the primary causative agent of tinea capitis and tinea corporis among Japanese martial arts athletes, particularly judo and wrestling practitioners in Japan. Recent epidemiological surveys on dermatophytosis in Japan have identified *T. tonsurans* as the most common causative pathogen of tinea capitis, accounting for 42.1 % of cases [1]. In contrast, a 2024 review from China reported that *Microsporum canis* was the most frequent causative agent of kerion celsi, responsible for 48.8 % of all cases, while *T. tonsurans* accounted for only 5.1 % [2]. Accurate diagnosis of tinea capitis is crucial, because it is often misdiagnosed as eczematous dermatitis, and inappropriate use of topical steroids may lead to progression to kerion celsi.

The diagnosis of tinea capitis requires the identification of fungal elements through KOH (potassium hydroxide) direct microscopy or fungal culture, but histopathological examination is also valuable. The observation of inflammatory destruction of hair follicles is crucial for differentiating kerion celsi, and the identification of the fungal invasion pattern in hair can aid in determining the causative dermatophyte species. Hair invasion is classified into endothrix (fungal presence within the hair shaft, e.g., *T. tonsurans*) and ectothrix (fungal presence on the hair surface, e.g., *M. canis*) [3]. Treatment requires selecting systemic antifungal agents based on the causative pathogen. The therapeutic efficacy of antifungal drugs may vary depending on whether the infection is endothrix or ectothrix [4,5]. Specifically, oral terbinafine is recommended for infections caused by *Trichophyton* species, which induce endothrix infections, whereas oral griseofulvin or itraconazole is recommended for infections caused by *Microsporum* species, which induce ectothrix infections [4]. Topical antifungal agents are not considered first-line treatments, as they do not sufficiently penetrate the infected hair shafts. However, antifungal shampoos containing ketoconazole, selenium sulfide, or ciclopirox are sometimes used as adjuncts to systemic therapy to reduce shedding of fungal spores, prevent reinfection, and maintain scalp hygiene [6,7].

During the treatment of inflammatory dermatophytosis, there is a risk of developing dermatophytid reactions, which can be mistaken for drug eruptions. Dermatophytid reactions commonly present as bilateral and symmetrical eruptions in the early phase of treatment, with no fungal elements detected in the lesions. These reactions usually resolve as the primary lesions improve or heal. However, misdiagnosing dermatophytid reactions as drug eruptions and discontinuing systemic antifungal therapy may lead to inadequate treatment [8], potentially causing scarring and alopecia. Therefore, in the acute phase, it is crucial to distinguish between dermatophytid reactions and drug eruptions to determine whether to continue, adjust, or discontinue antifungal therapy.

We report a case of kerion celsi caused by *T. tonsurans* accompanied by a dermatophytid reaction. The reaction was initially suspected to be a drug eruption, but it was diagnosed as a dermatophytid reaction, and oral antifungal therapy was continued, leading to clinical improvement. Furthermore, histopathological examination confirmed endothrix invasion in transverse sections of infected hairs, aiding in the definitive diagnosis and identification of the causative species.

## 2 Case presentation

A 16-year-old male high school judo athlete presented with hair loss in the occipital region, which had begun one month prior to his initial visit (day −30). He had no significant past medical history and had been practicing judo for over five years. Two weeks prior to visiting our institution, he was diagnosed with tinea capitis at another clinic based on the identification of fungal hyphae via KOH direct microscopy (day −11). Treatment with oral terbinafine hydrochloride and topical ketoconazole cream was initiated. However, one week later, his condition worsened, with the development of abscesses and infiltration in the hair loss area, as well as erythematous papules on his limbs and trunk. Suspecting a drug eruption due to antifungal agents, he was referred to our department.

**Fig. 1:**
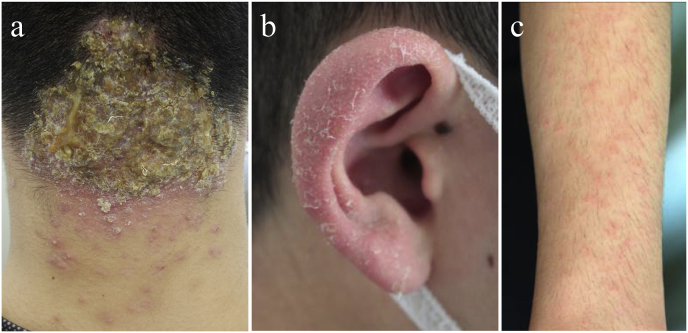
Clinical features. a: A 7 × 5 cm hair loss lesion with purulent discharge and crusts on the occiput, with some areas showing black dots. b: Scales were observed on both auricles.c: Symmetrical pruritic erythematous papules were observed on the chest and both forearms.

At the initial visit (day 0), a 7 × 5 cm alopecic area with purulent discharge, crusting, and black dots (black-dot tinea capitis) was observed in the occipital region. Bilateral cervical lymphadenopathy and multiple erythematous papules, scales, and pustules were noted around the hair loss area. KOH direct microscopy of the scales confirmed fungal hyphae. Scales were also observed on both auricles, and pruritic, erythematous papules were symmetrically present on the chest and forearms. However, KOH microscopy of these lesions revealed no fungal elements (Fig. 1). The patient was afebrile, with a stable general condition. Laboratory results revealed mild leukocytosis (13,680/μL; neutrophils 74.6 %, eosinophils 2.6 %) and elevated C-reactive protein (CRP) levels (1.69 mg/dL).

**Fig. 2:**
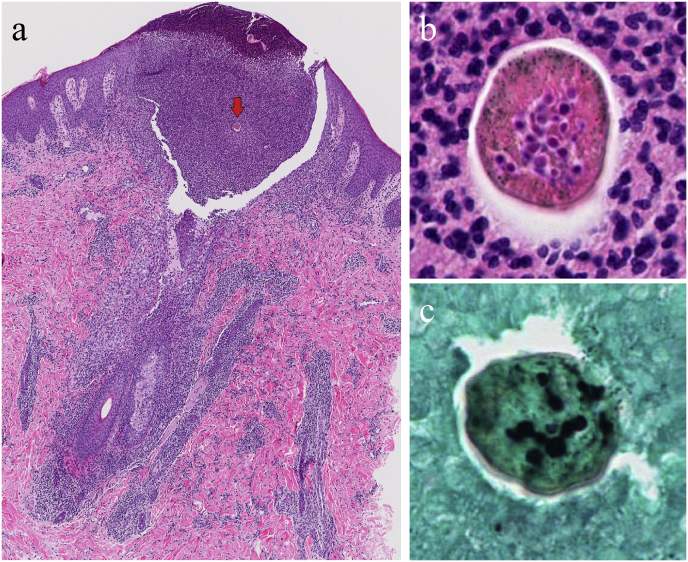
Histopathological findings. a: Infiltration of neutrophil-predominant inflammatory cells was observed around the dermal hair follicle. While the structure of the dermal hair follicle was preserved, the epidermal side was replaced by a pustule containing a transverse section of the affected hair (arrow) (HE, × 100). b: Magnified view of the transverse section of the infected hair. Fungal hypha-like structures are observed in the hair shaft (HE, × 400). c: Fungal elements stained black with Grocott stain were observed in the hair shaft, and endothrix infection is confirmed in the transverse section (GMS, × 400).

A skin biopsy was performed from the abscess at the margin of the alopecic lesion (day 0). Histopathology revealed inflammatory cell infiltration, predominantly neutrophils, around hair follicles, with preservation of follicular architecture. Neutrophil-rich abscesses were observed at follicular openings, along with transverse sections of infected hairs. Fungal hyphae were identified within the hair shafts using hematoxylin and eosin (HE) staining and Grocott's methenamine silver (GMS) staining, confirming endothrix invasion with large spore formation (day 5) (Fig. 2).

**Fig. 3:**
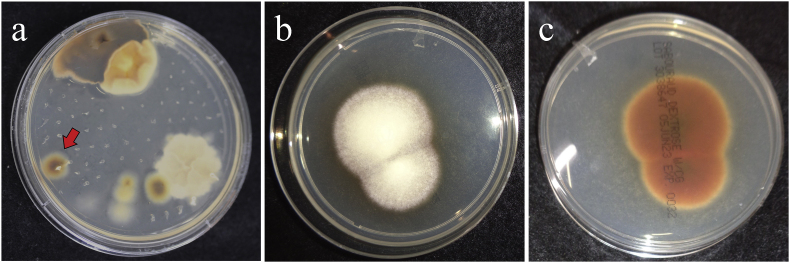
Fungal Culture. a: A fungal culture was performed by scraping the patient's scalp with a hairbrush. This image shows the reverse side of the medium after 12 days of incubation at room temperature. Various colony morphologies were observed, but colonies with a reddish-brown reverse (indicated by the arrow) were selected and subcultured. b: Subcultured colony from “Fig. 3-a”. After 20 days of incubation at room temperature on Sabouraud agar medium, the colony surface exhibited a white, cotton-like texture. c: The reverse side of the isolated colony exhibited reddish-brown pigmentation.

Fungal cultures were conducted using a hairbrush sample from the occipital lesion. After 14 days of incubation at room temperature (day +14), five colonies, including contaminants, were identified. A colony with a white powdery surface and reddish-brown reverse side was isolated and further cultured (day +20) (Fig. 3). Molecular analysis of the isolate (KMU11014) performed at Kanazawa Medical University confirmed 100 % identity with the internal transcribed spacer (ITS) sequence of the *T*. *tonsurans* reference strain (CBS 496.48; GenBank accession number NR_144891.1). Antifungal susceptibility testing revealed no differences compared to the reference strain (Table 1). However, identification to the species level was not possible using MALDI-TOF MS (Matrix-Assisted Laser Desorption/Ionization Time-of-Flight Mass Spectrometry) version 3.0.

**Table 1:** MIC values of the detected fungi (KMU 11014).

	Control	KMU 11014
	*T. mentagrophytes*	*T. tonsurans*
	PDA	PDA
↓CLSI_M38ed3	30 °C, 96h	30 °C, 96h
Terbinafine	0.015	0.004
Itraconazole	0.015	0.004
Luliconazole	0.00025	0.00006
Ravuconazole	0.03	0.004
Lanoconazole	0.001	0.00025

Methodology: According to CLSI (Clinical and Laboratory Standards Institute) Standard.

Based on these findings, the patient was diagnosed with kerion celsi caused by *T. tonsurans*, and continued systemic antifungal therapy was deemed necessary.

The patient presented to our hospital with suspected drug eruption. Although a dermatophytid reaction was included in the differential diagnosis, drug eruption could not be completely ruled out at that time. As a precaution, oral terbinafine hydrochloride and topical ketoconazole cream were discontinued on day 0, pending the results of a skin biopsy and blood tests. Topical steroids were applied to the erythematous papules on the limbs, which resolved within a week. By day +10, a diagnosis of kerion celsi caused by *T*. *tonsurans* was confirmed. However, the initial lesion on the occipital area showed no improvement. This prompted the decision to initiate systemic antifungal therapy. After discussions with the patient's parents, itraconazole capsules (original drug, 100 mg/day) were prescribed as an alternative to terbinafine. Topical treatment was switched to miconazole nitrate shampoo.

After nine weeks, hairbrush cultures yielded negative results, and treatment was completed. Five months later, hair regrowth was observed in the alopecic area without scarring (day +144). A drug lymphocyte stimulation test (DLST) for terbinafine hydrochloride, conducted four months after the initial visit (day +126), was negative.

Considering the absence of eosinophilia, the atypical presentation of erythematous papules compared to known terbinafine-induced eruptions (e.g., erythema multiforme, toxic epidermal necrolysis/Stevens-Johnson syndrome, fixed drug eruption, and acute generalized exanthematous pustulosis), as well as the negative DLST result, we concluded that the reaction was not a terbinafine-induced drug eruption.

## 3 Discussion

The distinguishing features of our case include confirmation of transverse sections of infected hair with endothrix-type parasites in histopathological examinations and presence of a dermatophytid reaction at the initial visit to our department.

**Fig. 4:**
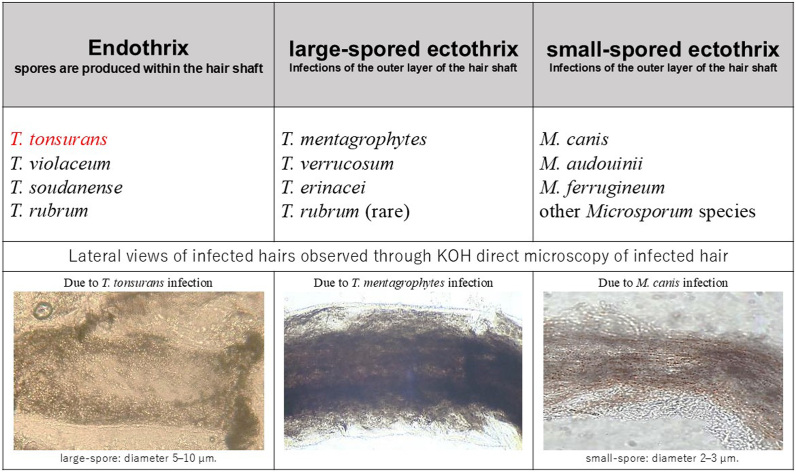
Classification of dermatophyte species showing ectothrix and endothrix infection and their direct microscopic images. It is challenging to visually distinguish between ectothrix and endothrix infections in lateral views of infected hairs observed through KOH direct microscopy. This figure is partially modified and referenced from Mateja Dolenc Voljč's 'Dermatophyte Infections in Humans: Current Trends and Future Prospects [17].

The parasitic forms of dermatophytes in hair are classified into endothrix and ectothrix types based on the pattern of hair invasion by the pathogens, which aids in fungal species identification. Endothrix infections, where spores are produced within the hair shaft, are typically observed with dermatophytes of the *Trichophyton* genus, such as *T. tonsurans*, *T. violaceum*, and *T. soudanense*. In contrast, ectothrix infections, characterized by spore production on the outer surface of the hair shaft are generally associated with dermatophytes of the *Microsporum* genus, such as *M. audouinii* and *M. canis* [9]. However, distinguishing between endothrix and ectothrix invasion based on two-dimensional lateral views of infected hair observed using KOH direct microscopy is difficult (Fig. 4). The difference becomes evident through observation of transverse sections of infected hair in deep-cut histopathological sections, enabling identification of the pathogen. Intrapilary hyphae fragment into arthroconidia up to 8 μm in diameter, which are entirely contained within and completely fill the hair shaft [3]. From this perspective, the value of histopathological diagnosis in this condition is significant. Elmas et al. emphasized the importance of histopathological diagnosis in the differential diagnosis of tinea capitis, noting that detecting fungal elements is not always possible [10]. Bourdages et al. reported a detection rate of infected hair in histopathological samples of fungal folliculitis at 54.2 % (IQR 33.3 %, 100.0 %) [11]. Although histopathological diagnosis is a useful diagnostic tool, careful selection of biopsy sites is crucial, along with creating serial sections and using PAS (Periodic Acid-Schiff)/GMS staining to prevent false negatives.

Regarding the complication of tinea known as dermatophytid (or trichophytid) reaction, it occurs when tinea rapidly worsens for some reason, leading the patient to develop an allergic state to dermatophytes or their metabolic products, resulting in sterile eczematous reactions on distant skin areas. The reported incidence of dermatophytid reactions varies. Grappel et al. reported an incidence of 4.2 % in children and 4.6 % in adults, with a male-to-female ratio of 1:1. The primary lesion was located on the scalp in children and on the feet in adults [12]. Niels et al. reported the incidence of dermatophytid reactions by fungal species, noting that 9 of 128 (7 %) patients with tinea pedis caused by *T. rubrum* developed dermatophytid reactions, while 27 of 78 (35 %) patients with tinea pedis caused by *T. mentagrophytes* developed dermatophytid reactions [13]. Dermatophytid reactions have been reported with various antifungal agents. Mayser observed that these reactions are particularly common with newer, more effective antifungal agents, including fluconazole, itraconazole, and terbinafine [14]. Clinically, differentiating dermatophytid reactions from drug eruptions is vital, especially when tinea lesions develop during antifungal therapy. Terbinafine may rarely induce acute generalized exanthematous pustulosis (AGEP) or pustular psoriasis, but these clinical findings differ from those of dermatophytid reactions [15].

Recently, there has been an increase in clinical and basic research reports regarding antifungal resistance. Drug-resistant strains of *T. rubrum*, *T. mentagrophytes*, *Candida auris*, and *Aspergillus fumigatus* have become significant problems. However, according to Futatsuya's report, no drug-resistant strains of *T. tonsurans* have been detected in Japan [16].

According to Elmas, the average time required to diagnose adult tinea capitis is 8–10 months, with 8.5 % of patients taking over a year to receive a diagnosis [10]. Delayed definitive diagnosis may lead to misdiagnosis as eczematous dermatitis, resulting in incorrect treatment and progression to kerion celsi, which can cause scarring or alopecia post-treatment. Therefore, managing this condition requires clinical examinations, including histopathological testing for fungal species identification and accurate diagnosis based on these findings. Even in cases where dermatophytid reactions occur, it is unnecessary to interrupt treatment for tinea, as the lesions will naturally resolve with continued use of antifungal agents.

It is important to differentiate dermatophytid reactions from drug eruptions to avoid unnecessary discontinuation of antifungal therapy.

## CRediT authorship contribution statement

**Makoto Ishiai:** Writing – review & editing, Writing – original draft, Visualization, Resources, Investigation, Data curation. **Hiroshi Tanabe:** Writing – review & editing, Writing – original draft, Visualization, Supervision, Resources, Project administration, Methodology, Investigation, Data curation, Conceptualization.

## Ethical form

This study received no funding, and there are no potential conflicts of interest to declare. We obtained written and signed consent to publish the case report from the patient.

## Conflict of interest

There are none.
